# Supplementary material for: diaTracer enables spectrum-centric analysis of diaPASEF proteomics data
Source: Nat Commun. 2025 Jan 2;16:95. doi: 10.1038/s41467-024-55448-8 (PMC11696033; doi:10.1038/s41467-024-55448-8)
Supplement: Supplementary file 2 — Description of Additional Supplementary Files [file 41467_2024_55448_MOESM2_ESM.pdf]

Description of Additional Supplementary Files:

Supplementary Data 1: Proteins containing semi-tryptic peptides quantified by FragPipe semi-tryptic workflow from the CSF dataset. The first column is the protein entry name. The second column is the number of semi-tryptic peptides quantified in the protein. The third column is the number of tryptic peptides quantified in the protein. The fourth column is the ratio between numbers of quantified semi-tryptic and tryptic peptides. The following columns are attributes of proteins from Uniprot.

Supplementary Data 2: Proteins containing semi-tryptic peptides mapping to the region immediately following the signal peptides in the N-terminal portion.

Supplementary Data 3: Protein differential expression results downloaded from FragPipe-Analyst for FragPipe tryptic (Sheet 1) and FP-diaTracer semi-tryptic (Sheet 2) workflow. The adjusted p-value is from the Moderated two-sided t-test followed by the Benjamini-Hochberg procedure.
